# Morbus Coxalgia, or Hip Disease

**Published:** 1854-10

**Authors:** R. L. Hale

**Affiliations:** Dundee, Kane Co. Ill.


THE NORTH-WESTERN

MEDICAL AND SURGICAL JOURNAL.

NEW SERIES.

VOL. III. OCTOBER, 1854. NO. 10.

### ORIGINAL COMMUNICATIONS.

ART. I.—*Morbus Coxalgia, or Hip Disease.*—By R. L. Hale, M.D., Dundee, Kane Co. Ill.

March, 15th, 1854, was called to see a patient aged 3 years, suffering with what the parents supposed to be a dislocation of the hip. I learned from the parents that three weeks previous to my visit, they first noticed the inability of the child to use one leg; and that every effort to compel her to bear weight on it caused her to cry out with pain. The child had been about, and played with other children as usual, and they were not aware that it had received any mechanical injury. At the present time the hip is swollen, to almost double its natural size, and it is quite tender to pressure, firm and unyielding to the touch with the temperature considerably increased. The position of the foot and the length of the limb, so far as could be ascertained by ordinary measurement, were natural. The whole limb was moderately flexed, and would admit of only a slight degree of motion in any direction without producing great pain. There was no swelling or lameness in other joints, and very little febrile action or other constitutional disturbance. I could not learn that the parents had ever been affected with either scrofulous, eruptive, or rheumatic affections; and the child presented no marks of a scrofulous diathesis. Having but a small amount of experience in diagnosticating and treating diseases like the present one, I was in much doubt as to its true nature. The suddenness of the attack with the robust appearance of the child, and the rapid increase of the swelling, seemed to preclude the idea of its being coxalgia or ordinary hip disease. If it was rheumatic inflammation, we should expect with so much swelling, and pain while the patient is at rest, more redness, and more general febrile action. While the correct position of the foot, and length of the limb, with the absence of any fall or other mechanical injury, equally precluded the idea of a dislocation of the head of the femur. I confess that I left the patient with many doubts in regard to the true nature of the disease.

On making a second visit and finding no material change in the patient, with a strong inclination on the part of the parents to regard it as a dislocation, I thought it best to follow David Crocket's rule, "be *sure you are right* and then go ahead." So with the consent of the parents, I started with them and the child for Chicago, by railroad; at which place we arrived in safety, and obtained the council of Prof N. S. Davis and Dr. J. W. Freer. After a careful examination they agreed in the opinion that the disease was a low grade of inflammation affecting chiefly the *fibrous tissues* surrounding the hip-joint. Prof. Davis thought the child bore the marks of a scrofulous temperament, and expressed the fear that if not soon removed the disease would extend into the joint and constitute true coxalgia. He suggested the propriety of keeping the patient at rest; applying leeches to the hip every second day, with fomentations of aconite leaves during the intermediate time, until the heat, tenderness, and swelling were materially diminished, and then cover the hip with a plaster of Ammoniaco Cum Hydrarg, and a little below it keep open a blistered surface or issue. Internally, he advised the use of mild mercurial alteratives for a few days, followed by the sat. tinct. of cimicifuga rasemosa root and iodide of potassa for a considerable time. The patient returned home and the foregoing suggestions were strictly carried out. After leeching and fomenting the hips for several days, I first applied a blister over the part. When this healed I covered the whole hip with the emplastrum ammoniaco cum hydrarg., and made an issue just below the plaster. This and the plaster were continued 8 weeks. When I first began the use of the cimicifuga, I united with it the vinum colchici, but the latter acted so much on the bowels that I had to discontinue it And at times the cimicifuga and iodide of potassa caused so much action of the bowels as as to require opiates to restrain them. Still their use was steadily continued for eight weeks, when the patient had entirely recovered, and is up to the present time quite well.

*Query.*—Was not the *nature* of the disease *rheumatic* inflammation of fibrous tissues around the hip joint ?

*Case* 2.—I was called a few days since to see a little girl about eight years of age, who was reported to have had dislocation of the hip joint several months previous.

I found the girl slender, and delicate in appearance, the skin thin and pale, the hair fine and light colored, the eye clear and bright, with a pulse slightly accelerated, and slight febrile exacerbations each night, with disturbed sleep.

She would not put the right foot down upon the floor. or bear the slightest weight on it. She could bear to have the foot turned in either direction, and would permit a very moderate movement of the limb in any direction, but would not permit it to be either fully flexed or extended. Neither would she bear more than a slight abduction.

By careful measurement, I could detect no shortening of the limb, neither did it appear much wasted; but the gluteal region seemed flattened more than the opposite side

There was, however, no swelling, and no tenderness except when pressure was made on the trochanter major. Pressure made on the trochanter, or on the knee or the sole of the foot, in such a way as to move the head of the femur in its socket, always gave much pain. The mother gave me the following history of the case, viz.: Nearly one year since the girl fell from a wagon, striking on the side and hip. She was helped up, and after a few minutes could walk with only a slight limp. This slight limping, with a complaint of soreness in the hip, continued until it caused the parents to be uneasy about it, although it was at no time so great as to prevent the child from running about freely. Six weeks after the fall they consulted a physician, who decided that the head of the femur was dislocated, and went through with the usual performance of reducing it. Considerable force was used for extension and counter-extension, and the doctor claimed that the bone was reduced. No change, however, followed the operation, except a moderately increased soreness and lameness, which passsed away again in a few days, leaving precisely the same lameness as before.

The disease or difficulty in walking continued with but little change until but a few weeks since, when the patient again accidentally fell from a cradle or little wagon; since which time she has been wholly unable to bear any weight on the right foot, or even to put it down upon the floor. Feeling some hesitation about deciding on the nature of the case after the previous diagnosis, which had been made in reference to dislocation of the hip, I took this patient also to Prof. Davis, of Chicago. After examining the patient, and eliciting a full history of the case, as detailed above, he expressed his conviction that the case was clearly one of slow disease within the hip joint, induced, in the first instance, by a fall on the hip, which excited a low grade of inflammatory action in the joint. The child being of a scrofulous habit, the morbid action set up in the synovial membrane and cartilages of the joint continued only in a very moderate degree, until the second and more recent fall greatly increased its intensity and progress.

The prognosis was, of course, much more unfavorable than in the first case related. The treatment, with its results, may be given hereafter.

